# Vascular Supply to the Liver: A Report of a Rare Arterial Variant

**DOI:** 10.1155/2013/969327

**Published:** 2013-09-18

**Authors:** Peter B. Johnson, Shamir O. Cawich, Sundeep Shah, Michael T. Gardner, Patrick Roberts, Brian Stedman, Neil W. Pearce

**Affiliations:** ^1^Section of Radiology, Department of Surgery, Radiology, Anaesthetics and Intensive Care, Faculty of Medical Sciences, University of the West Indies, Mona Campus, Kingston, Jamaica; ^2^Department of Clinical Surgical Sciences, Faculty of Medicine, University of the West Indies, St. Augustine Campus, St. Augustine, Trinidad and Tobago; ^3^Section of Anatomy, Basic Medical Sciences, University of the West Indies, Mona Campus, Kingston 7, Jamaica; ^4^University Surgical Unit, Southampton General Hospital, Southampton SO16 6YD, UK

## Abstract

In the classic description of hepatic arterial supply, the common hepatic artery originates from the coeliac trunk. However, there are numerous variations to this classic pattern. We report a rare variant pattern of hepatic arterial supply and discuss the clinical significance of this variation.

## 1. Introduction

In the classic description of the arterial supply to the liver, the coeliac trunk trifurcates into left gastric, splenic, and common hepatic arteries [1–5]. The common hepatic then bifurcates at its termination into the proper hepatic artery and gastroduodenal arteries [1–5]. However, there are numerous variations to this classic pattern.

Michels [6] first described variants of the classic anatomy of the hepatic arteries in 1953. Based on a series of cadaveric dissections, Michels [7] then proposed a classification system that described ten anatomic variants. The classification is in common use to describe variant hepatic arterial branching patterns and allows standardization of anatomic descriptions [8]. We report a variant that is not described by the Michels' classification [7].

## 2. Case Report

A 59-year-old female patient with a diagnosis of locally advanced invasive ductal carcinoma of the left breast was referred for a staging CT scan of the abdomen and pelvis. The scan was done using a Philips Brilliance 64 slice multidetector CT scanner. Nonionic contrast media (Ultravist 300) in a volume of 100 mLs were administered via pressure injector at a rate of 3.5 mL/min. The liver was found to be normal; however, she had evidence of metastases to the spleen, several vertebrae, and the pelvis.

**Figure 1:**
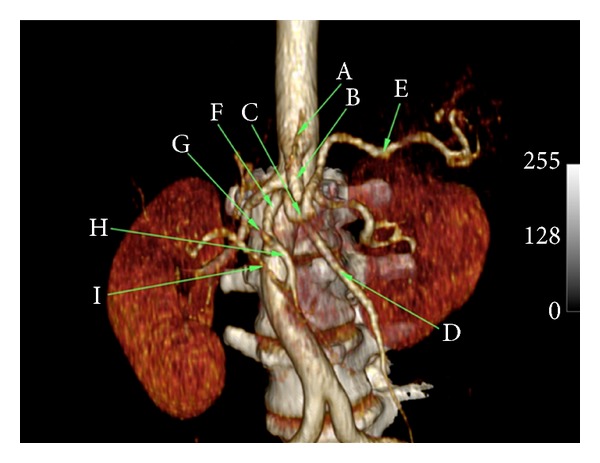
Reconstructed coronal CT images demonstrating the new anatomic variant. Key: left gastric artery—A; celiacomesenteric trunk—B; coeliac trunk—C; superior mesenteric artery—D; splenic artery—E; common hepatic artery—F; left hepatic artery—G; gastroduodenal artery—H; right hepatic artery—I.

**Figure 2:**
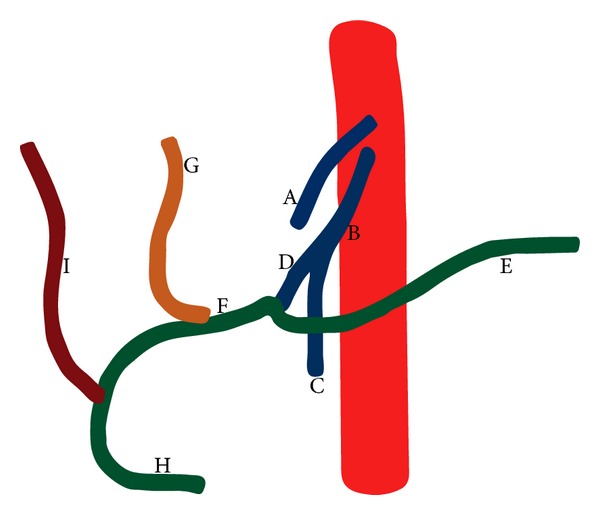
Illustration of the anatomic variant. Key: left gastric artery—A; celiacomesenteric trunk—B; superior mesenteric artery—C; coeliac trunk—D; splenic artery—E; common hepatic artery—F; left hepatic artery—G; gastroduodenal artery—H; right hepatic artery—I.

**Figure 3:**
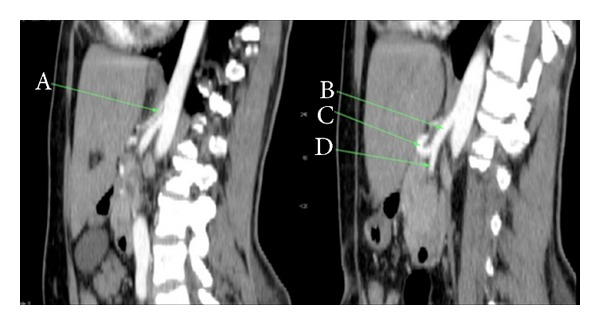
Sagittal reconstructed CT images demonstrating the new anatomic variant. Key: left gastric artery—A; unnamed common trunk—B; coeliac trunk—C; superior mesenteric artery—D.

An incidental finding of abnormal arterial branching was noted at the upper abdominal aorta (Figures 1 and 2). The left gastric artery originated directly from the anterior surface of the abdominal aorta shortly after it entered the abdomen through the diaphragmatic hiatus. Thereafter, it followed its normal course along the lesser curvature of the stomach. At the level of the first lumbar vertebra, there was a large arterial trunk originating from the anterior surface of the aorta, consistent with the celiacomesenteric trunk described by Ishigami et al. [9]. After coursing 2.5 cm, the celiacomesenteric trunk bifurcated into the superior mesenteric artery and the coeliac trunk that was unusually long and tortuous (Figure 3). The splenic artery coursed to the left over the superior mesenteric artery and vein toward the splenic hilum where it divided into segmental arteries to supply the spleen in normal fashion. The left hepatic took an early origin directly off the common hepatic artery and travelled up toward the hilum in a plane superficial to the portal vein but in a more medial position than usual. The right hepatic artery took its origin from the distal gastroduodenal artery behind the pancreatic head to course superiorly in the free end of the gastroduodenal ligament, posterolateral to the portal vein. The bile ducts were normal in calibre and were not well visualized on CT scans. At the hepatic hilum, the left and right hepatic arteries branched in the usual fashion to supply the liver that was divided into conventional hepatic segments.

## 3. Discussion

Michels' classification proposed ten anatomic types to describe all possible variations in hepatic arterial supply [7]. The anatomic variant encountered here is not described by the Michels classification [7]. It is important to appreciate the variant because these patients are at high risk for inadvertent injury during dissections in hepatobiliary and pancreatic operations. Inadvertent injury could result in disastrous complications such as liver ischaemia, anastomotic leaks, biliary strictures, and haemorrhage [2–5].

This highlights the need for routine evaluation of vascular anatomy with CT angiography and/or magnetic resonance angiography in all patients undergoing elective hepatobiliary and pancreatic interventions [10–14]. Preoperative knowledge of variant arterial anatomy has the potential to reduce operative morbidity and mortality by providing an intraoperative roadmap [10–13]. It is also required to plan endovascular therapies such as transarterial embolization for hepatic malignancies [15–17]. This is further supported by the fact that variations to the classic arterial supply to the liver are present in 37% of unselected persons in the Caribbean population [8].

These variations hold clinical significance to radiologists and surgeons who perform invasive hepatobiliary and pancreatic procedures. In these cases modification of the operative procedure may be required with planned arterial reconstruction and modified patient consent to reflect the increased perioperative risk [8].

## 4. Conclusion

Although the classic pattern of arterial supply to the liver describes the common hepatic artery originating from the coeliac trunk, there are numerous variations to this classic pattern. The common trunk encountered here is a rare variant that is not included in Michels' classification of arterial variations. It is important that clinicians are aware of these variations because they carry clinical significance.
